# Use of Farnesyl Transferase Inhibitors in an Ageing Model in *Drosophila*

**DOI:** 10.3390/jdb11040040

**Published:** 2023-10-29

**Authors:** Annely Brandt, Roman Petrovsky, Maria Kriebel, Jörg Großhans

**Affiliations:** 1 Bieneninstitut Kirchhain, 35274 Kirchhain, Germany; 2 Department of Biology, Philipps University, Karl-von-Frisch-Straße 8, 35043 Marburg, Germany

**Keywords:** HGPS, *Drosophila*, farnesyl transferase inhibitor, ABT-100, nuclear envelope, lamin, kugelkern

## Abstract

The presence of farnesylated proteins at the inner nuclear membrane (INM), such as the Lamins or Kugelkern in *Drosophila*, leads to specific changes in the nuclear morphology and accelerated ageing on the organismal level reminiscent of the Hutchinson–Gilford progeria syndrome (HGPS). Farnesyl transferase inhibitors (FTIs) can suppress the phenotypes of the nuclear morphology in cultured fibroblasts from HGPS patients and cultured cells overexpressing farnesylated INM proteins. Similarly, FTIs have been reported to suppress the shortened lifespan in model organisms. Here, we report an experimental system combining cell culture and *Drosophila* flies for testing the activity of substances on the HGPS-like nuclear morphology and lifespan, with FTIs as an experimental example. Consistent with previous reports, we show that FTIs were able to ameliorate the nuclear phenotypes induced by the farnesylated nuclear proteins Progerin, Kugelkern, or truncated Lamin B in cultured cells. The subsequent validation in *Drosophila* lifespan assays demonstrated the applicability of the experimental system: treating adult *Drosophila* with the FTI ABT-100 reversed the nuclear phenotypes and extended the lifespan of experimentally induced short-lived flies. Since *kugelkern*-expressing flies have a significantly shorter average lifespan, half the time is needed for testing substances in the lifespan assay.

## 1. Introduction

The nuclear lamina is a network of lamin polymers and lamina-associated proteins, which provides mechanical support, controls the size and shape of the nucleus, and mediates the attachment of chromatin to the nuclear envelope. A range of human disorders can be linked to defects in the components of the nuclear lamina [1,2,3], of which some have been linked also to physiological ageing [4,5,6,7,8]. Mutations in the human *lamin A/C* gene (LMNA) cause at least eleven different human diseases called laminopathies [9,10,11,12], with Hutchinson–Gilford progeria syndrome (HGPS) as a prominent example [8,13,14,15]. In the majority of HPGS variants, the C-terminal farnesylation site of LMNA is affected, which results in the presence of a permanently farnesylated variant of Lamin A called Progerin [8,13,14,16]. Given the lipophilic farnesyl moiety, Progerin intrinsically associates with the membrane bilayer and interferes in some way with the integrity of the nuclear lamina and causes misshapen nuclei [2,8,13,14,17].

Even in healthy individuals, Progerin is present in aged human cells [4]. And thus may participate in physiological ageing. In healthy individuals, Progerin has been shown to accumulate during the lifetime in a subset of dermal fibroblasts [5], in coronary arteries [7], and in bone marrow stem cells [18]. The farnesylation of Progerin and the frequency of misshapen nuclei in HGPS cell can be suppressed by the inhibition of protein farnesylation with a protein farnesyl transferase inhibitor (FTI). It is well-established that FTIs prevent the accumulation of Progerin at the nuclear envelope and reduce nuclear deformation and ageing-like phenotypes [19,20,21,22,23,24,25]. FTIs have been tested in clinical trials for the treatment of HGPS patients [26,27].

Remarkably, the induction of ageing-like phenotypes by the expression of farnesylated lamina proteins is observed also in other vertebrates [28] and invertebrates [29,30,31,32,33,34,35]. Furthermore, laminopathy-related phenotypes can be induced by other farnesylated nuclear proteins beside Progerin. Lamin B variants, or the insect-specific Kugelkern (Kuk), can induce ageing-like phenotypes in *Drosophila* [29,30]. Besides the C-terminal farnesylation site, *Drosophila* Kuk shares no apparent sequence homology with lamins. The expression of Kuk or Lamin B or farnesylated variants in vertebrate cells induces nuclear lobulation and deformations in conjunction with DNA damage, and changes in histone modifications [29,36,37,38,39] reminiscent to those found in HGPS cells or in cells from aged human individuals [4]. Lobulated, wrinkled nuclei are characteristic for the premature ageing syndrome HGPS, but are also present in healthy ageing humans, in nematodes, and in *Drosophila* [4,29,30,31,40]. In wild type adult *Drosophila* flies, age-related changes in muscle morphology and physiology are observed [41]. This involves increased size and aberrant morphology in aged flight muscle nuclei [29]. Such ageing-like phenotypes can be prematurely induced by the expression of farnesylated lamina proteins. The overexpression of Lamin B or Kuk induces aberrant nuclear shapes early in adult life and reduces the lifespan of the flies. The shorter lifespan correlates with an early decline in age-dependent locomotor behavior [29]. Thus, the lobulation of the nuclear membrane induced by the insertion of farnesylated nuclear proteins is associated with premature ageing-like phenotypes in cultured mammalian cells as well as in adult *Drosophila* [29].

One of the major obstacles on the way to develop new genetic interventions or drug treatments that concern ageing or prolong lifespan is that screens are time-consuming and limited in scale. By complementing existing and well-established assays with cultured cells with lifespan assays in adult *Drosophila* flies, we developed a relatively fast screening system for the identification of genes or substances that ameliorate ageing-like phenotypes and potentially prolong the lifespan [29]. To demonstrate the feasibility but also the limitations of our combined method, we employed two well-characterized inhibitors that interfere with the farnesylation of proteins: ABT-100 (Abbott, Chicago, IL, USA) and Simvastatin (Sigma, St. Louis, MO, USA) [42,43,44]. ABT-100 is an orally bioavailable farnesyl transferase inhibitor. Simvastatin is a specific inhibitor of HMG-CoA reductase, an enzyme involved in farnesyl biosynthesis.

## 2. Materials and Methods

### 2.1. Cell Culture

NIH3T3 cells were cultured in DMEM (Thermo Fisher Scientific/Invitrogen, Waltham, MA, USA supplemented with 10% FBS and 2 mM L-glutamine at 37 °C. We plated NIH3T3 cells in 6 wells containing cover slips and transiently transfected them with Effectene (Qiagen, Hilden, Germany) when they reached a confluence of 25–30% with pCS2HAkuk ([38], referred to here as *kuk*), pEYFP-Progerin or pCS2XlaminB2ΔNGFP ([36], referred to here as laminB2ΔN) (2 µg construct/6-well). The efficiency of transfection was generally at least one third. The YFP-Progerin plasmid was generated by cloning human Progerin (human Prelamin A with a 50 aa deletion) into the BspE1/XhoI sites of vector pEYFP-C1 (Takara Bio/Clontech, Mountain View, CA, USA).

### 2.2. Generating a Stable HeLa Cell Line with Inducible GFP-kuk Expression

GFP-kuk and GFP were cloned by PCR into the vector pBI.F3 [45], resulting in plasmids pBI-F3-GFP-Kuk and pBI-F3-GFP. The vector pBI.F3 allows for FRT/Flp-mediated insertion into the S/A locus in HeLa cells [45]. Targeted integration of the constructs was conducted as described by Weidenfeld [45]. Briefly, cells from the mother HeLa S/A cell line were plated in a six-well plate with standard medium except for tetracycline-free fetal calf serum (FCS) instead of FBS. The following day, the nearly confluent cells were transfected with 2 μg of pCAGGS-Flpe-IRES-Puro and 2 μg of targeting vectors (pBI-F3-GFP-Kuk, pBI-F3-GFP) using Effectene. The molar ratio of the plasmids was ~1:1. After overnight incubation, cells were transferred to a 10 cm Petri dish and Puromycin (5 μg/mL) was added for the selection of cells transfected with the pCAGGS-FLPe plasmid. After 36 h, the medium was changed and cells were selected with Ganciclovir (10–50 μM) for recombinants (loss of the HygTK-cassette). The medium was replaced by fresh selective medium every day for one week. Subsequently, ten clones were picked, amplified in selective medium, and induced with Doxycycline in order to confirm GFP-Kuk expression. Protein expression was checked by Western blotting and by immunofluorescence. The induction was performed by addition of 250 ng/mL Doxycycline to the medium. ABT-100 was used at a concentration of 6 μM. Cells were collected after 0–7 d and stained or lysed in Laemmli buffer in order to be used for Western blotting (0.5 × 10^5^ cells were loaded). Antibodies for Western blot: mAb414 (Sigma), mouse HP1α (Chemicon, Takahagi, Tokyo), RBBP (Abcam, Cambridge, UK), α-Tubulin (Sigma), Kuk [38].

### 2.3. Immunohistochemistry

Cells were cultivated for 48 h (HP1α and H3K9me3 staining) or for 72 h (H2A.X staining). After washing in PBS, cells were fixed with 2% formaldehyde in PBS containing 0.2% Tween and 0.5% NP-40 for 20 min at room temperature. After washing in phosphate-buffered saline (PBS), cells were permeabilized in PBS with 0.5% Triton X-100 plus 0.5% Saponin (Sigma) for 10 min. Consecutively, cells were blocked in PBS containing 0.1% Triton (PBT) with 5% BSA and stained in PBT containing primary antibodies, fluorescent secondary antibodies (4 µg/mL, Alexa, Molecular Probes, Eugene, OR, USA), or 4’,6’-Diamidin-2-phenylindol (DAPI), and mounted in Aquapolymount (Polyscience, Nieles, Il, USA). Antibodies for immunostainings: p-H2A.X (Chemicon, 1:5000), mouse HP1α (Chemicon, 1:2500), Kuk (0.2 µg/mL, [38]), H3K9me3 (Sigma/Upstate, 0.2 µg/mL).

### 2.4. Microscopy

To visualize the effect of the FTIs in vivo, adult males were anaesthetized. The head, abdomen, legs, and wings were cut off. The thorax was transferred to ice cold Schneider cell medium where it was split into half and mounted in 50% glycerol. For measurement of the muscle nuclei perimeters, we acquired digital fluorescent images of longitudinal adult muscles taken with a fluorescent microscope connected to a Progress camera and processed them with Photoshop (Adobe). Three independent experiments were analyzed by VassarStats (Mann–Whitney U-Test, Web-based VassarStats).

### 2.5. Drosophila Strains

Fly stocks were obtained from the Bloomington stock center [46], if not otherwise noted. Genetic markers, mutations and annotations are described in Flybase (http://flybase.org, accessed on 1 January 2020) [47]. GS-actin used the actin5C promoter [48], and GS-MHC used the myosin heavy chain promoter [49]. For cloning of pUASp-kuk, the cDNA from LD09231 was cloned as NotI-ApaI/blunt fragment into the NotI-XbaI/blunt sites of pUASp. UASp-GFP-kuk was generated by insertion of a GFP-encoding sequence at the start codon into pUASp-kuk. For the GFP-kuk and GFP-kukCS567 constructs, a sequence encoding GFP was inserted at the start codon, and a sequence of the C-terminus with the CS567 point mutation exchanged with the corresponding sequence in the plasmid pBKS-kuk^+^, which contains an 8.2 kb EcoRV-EcoRV fragment including the *kuk* locus [38]. For transgenesis, the GFP-kuk and GFP-kukCS567 constructs were transferred to the transformation vector Casper4. Transgenic flies were generated according to standard procedure with random transposase-mediated integration.

### 2.6. Lifespan Assays, Preparation of Muscle Tissue

All flies were raised and kept in a humidified, temperature-controlled incubator with 12 h on/off light cycle at 25 °C in vials containing standard cornmeal medium (2.5% yeast, 2.18% treacle, 1% soya meal, 8% cornmeal, 8% malt, 1.25% propionic acid). Flies were collected under short CO_2_-anaesthesia. Each demography cage was initiated with at least 100 newly eclosed males. The number of deceased flies was recorded every two to three days, when flies were transferred to fresh food plates. For induction with the GeneSwitch system, RU486 (Sigma) was added directly to the food to a final concentration of 200 µM. Food plates for control experiments contained an equal amount of the vehicles DMSO and ethanol as plates with RU486 or FTIs. The data across three to four replicate demography cages per treatment and genotype were combined. The concentration of ABT-100 was 1 µg/mL in the food. We did not systematically test a series of ABT-100 concentrations. Prism GraphPad and Sigmaplot software were used for survival data (log-rank test). Longitudinal muscles were prepared from young adult flies (2–7 d) heterozygous for GS-actin and UASp-GFP-kuk and fed for five days with food complemented with ABT-100 (1 µg/mL) or RU486 (200 µM). Living tissue was stained with DAPI and imaged with an epifluorescence microscopy. Muscles from at least three animals per genotype were analyzed.

## 3. Results

### 3.1. The Farnesyl Transferase Inhibitor ABT-100 Suppressed Ageing-like Phenotypes in Cultured Cells

Fibroblasts that express Progerin have been shown to develop phenotypes also found in fibroblasts from HGPS patients such as excessive folds of the nuclear envelope, an accumulation of unrepaired DNA damage, and reduced HP1α staining [17,50]. The suppression of these Progerin-induced phenotypes by FTIs, including dose–response relationships, are well established in cell culture [19,20,21,22,23,50,51] as well as on the organismal level in mice and HGPS patients [26,51,52].

**Figure 1:**
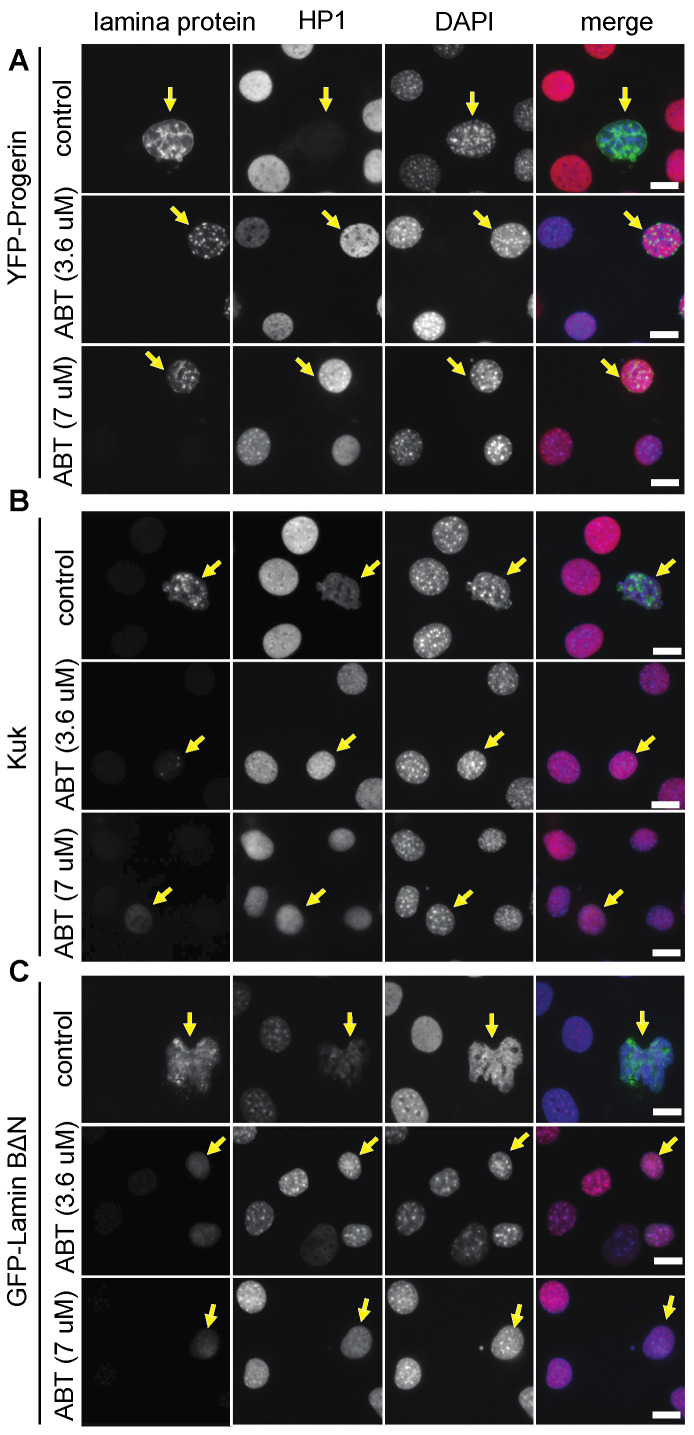
Ageing-like phenotypes are suppressed by ABT-100 treatment. NIH3T3 mouse fibroblasts transfected with farnesylated nuclear proteins (**A**) YFP-Progerin, (**B**) Kugelkern, or (**C**) GFP-LaminB2∆N and treated with ABT-100 at concentrations of 0 µm (control), 3.6 μM, or 7 μM, as indicated. Fixed cells were stained for (**A**) GFP, (**B**) Kuk, (**C**) LaminB (grey/green), HP1α (grey/red), and DAPI (grey/blue). Arrows point to nuclei of transfected cells. Scale bars 10 μm.

We first confirmed the activity of the FTI ABT-100 in our assay system with previously established ABT-100 concentrations [42,51,53]. When we treated Progerin-expressing NIH 3T3 fibroblasts with the farnesyl transferase inhibitor ABT-100, we observed a suppression of the nuclear phenotypes as expected (Figure 1, Table 1). Progerin was no longer concentrated at the nuclear envelope but was found in intranuclear spot-like accumulations. The nuclei appeared smooth and devoid of folds in the nuclear envelope, in contrast to the control cells with blebs and an abnormal nuclear shape. The HP1α staining intensity was restored to levels comparable to those of untransfected control cells (Figure 1A).

Fibroblasts expressing a truncated but constitutively farnesylated form of Lamin B, Lamin BΔN [36,37], showed a nuclear phenotype comparable to Progerin-expressing cells [29]. The transfected cells displayed foci and accumulations of the GFP-laminBΔN signal, strong nuclear envelope folds, and reduced HP1α staining (Figure 1C). When we treated those cells with ABT-100, we observed a suppression of these phenotypes: the nuclear envelope acquired a regular shape with few/no folds and HP1α staining was restored to normal levels. The GFP-laminBΔN signal was reduced to few spot-like accumulations and a diffuse GFP-laminBΔN signal was present throughout the nucleoplasm.

Kugelkern, a *Drosophila*-specific, farnesylated lamina protein, induces nuclear phenotypes comparable to Progerin or LaminBΔN [29,38]. The treatment with ABT-100 also suppressed the nuclear phenotypes in *kuk*-transfected fibroblasts (Figure 1B, Table 1). The GFP-kuk signal was no longer restricted to the nuclear envelope but was reduced to a diffuse signal throughout the nucleoplasm, indicating the efficient inhibition of farnesylation. In the ABT-100 treated cells, nuclear folds were absent and the HP1α-staining intensity was restored to levels comparable to those of the untransfected control cells (Figure 1B).

**Figure 2:**
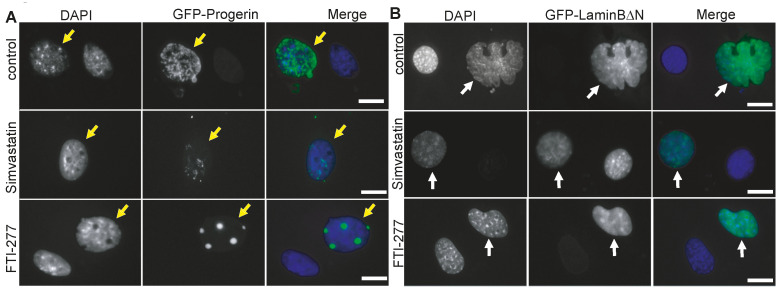
Simvastatin and FTI-277 suppress nuclear morphology phenotypes. NIH3T3 mouse fibroblasts transfected with farnesylated nuclear proteins (**A**) GFP-Progerin or (**B**) GFP-LaminB2∆N and treated with Simvastatin or FTI-277 as indicated. Fixed cells were stained for GFP (grey/green) and DAPI (grey/blue). Arrows point to nuclei of transfected cells. Scale bars 10 μm.

In addition to ABT-100, we tested a second FTI, FTI-277, and the statin Simvastatin, an established inhibitor of the enzyme hydroxymethylglutaryl-CoA reductase (HMG-CoA reductase), which catalyzes the entry step for the synthesis of cholesterol and other prenyl derivatives such as farnesyl residues. As expected from previous reports [43,44], both drugs reduced the GFP-Progerin signal at the nuclear envelope, and only a diffuse nucleoplasmic signal was observed (Figure 2, Table 2). The typical nuclear envelope malformations, like blebs or protrusions, were reduced by Simvastatin and FTI-277, and the nuclei had an overall normal shape (Figure 2A). Comparable phenotypes were observed in the cells expressing GFP-LaminBΔN (Figure 2B). The GFP-LaminBΔN signal was mostly nucleoplasmic. We observed an amelioration of the nuclear shapes with reduced blebs, lobulations, or nuclear envelope protrusions, albeit abnormalities were still present in the-drug treated cells. Consistent with previous reports, we conclude that the FTIs ABT-100 and FTI277 and Simvastatin were able to suppress the nuclear phenotypes induced by Progerin, Kuk, or Lamin BΔN expression in the cultured mouse fibroblasts.

### 3.2. Suppression of Kuk-Induced Nuclear Phenotypes by ABT-100 in HeLa Cells with Inducible kuk Expression

**Figure 3:**
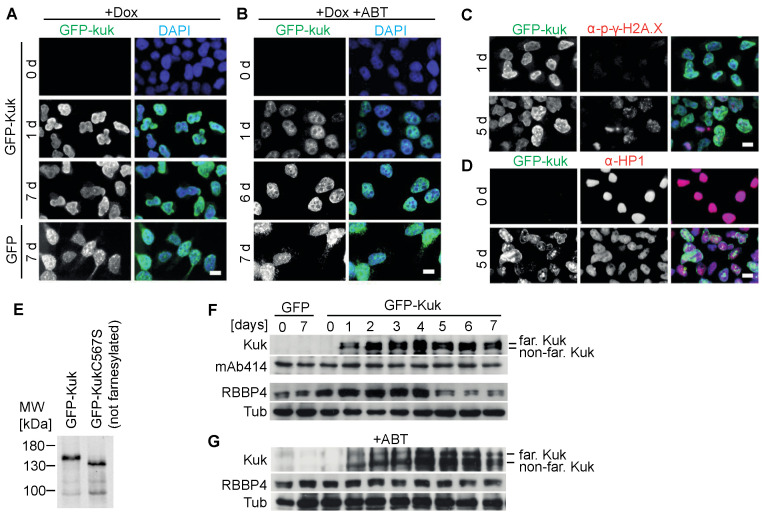
Stable GFP-kuk transfected Hela cells show abnormal nuclear shapes. Hela cell lines with integrated GFP-kuk or GFP constructs under control of a doxycycline (Dox) inducible promoter. GFP-kuk or GFP expression was induced by Dox for indicated periods of time. Cells were treated with ABT-100 as indicated. (**A**–**D**) Fixed cells stained for GFP (grey/green), phosphor-γ-H2A.X or HP1 (grey/red), and DAPI (grey/blue) as indicated. Scale bars 10 µm. (**E**) Western blots for Kuk with lysates from embryos expressing GFP-kuk or GFP-kukCS567 in a *kuk*-deficient background. The farnesylated form of Kuk is detected at a higher apparent molecular weight than the non-farnesylated form. (**F**,**G**) Western blots with lysates of Hela cells with induced GFP or GFP-kuk expression and ABT-100 treatment as indicated. The following proteins were detected: Kuk (with presumably farnesylated and non-farnesylated forms), nuclear pore antigens (mAB414), Nurd complex protein RBBP4, and α-tubulin (loading control).

To establish highly reproducible experimental conditions with inducible expression levels, we generated a Hela cell line with a genomic insertion of the construct, in which GFP-kuk expression can be induced by doxycycline treatment [45]. After 24 h of doxycycline induction, cells showed a prominent GFP-kuk signal at the nuclear lamina. The shape of the nuclei was deformed with large protrusions and internal folds (Figure 3A), similar to those observed in NIH 3T3 fibroblasts (Figure 1B) expressing *kuk*. The nuclei in the control cells expressing GFP remained round even after extended induction periods. The induction of GFP-kuk expression after one day and the increasing protein levels throughout the induction period were detected by Western blot analysis (Figure 3E). Two bands were detected, which represent the farnesylated (upper) and non-farnesylated forms of Kuk. The treatment of cultured S2 cells with FTI leads to a band shift in Western blots [54]. Furthermore, we assayed Kuk with Western blot in extracts from embryos homozygous for a *kuk* deletion (allele *kuk*Δ15, [38]) but expressing the GFP-kuk or GFP-kukCS567 transgene. In the CS567 mutation, the target cysteine residue of farnesylation was mutated to a serine residue. Comparing the two extracts, we detected a band shift towards a lower apparent molecular weight with the GFP-kukSC567 transgene (Figure 3E, Supplemental Figure S1), suggesting that the farnesylation of Kuk leads to a slower movement and higher apparent molecular weight in SDS-PAGE.

The ABT-100 treatment prevented the formation of abnormally shaped nuclei in GFP-kuk-expressing Hela cells (Figure 3B). Even after six days of induction, no prominent nuclear envelope folds were observed and the GFP-kuk signal was predominantly nucleoplasmic. In the Western blot analysis, a duplet of Kuk bands was detected. The lower band was more prominent after the ABT treatment than without the ABT-100 treatment (Figure 3F,G, Supplemental Figure S1), which would be consistent with a faster migration of the non-farnesylated form of Kuk in SDS-PAGE (Figure 3E) [54]. Taking together the loss of the nuclear envelope staining and the apparent band shift in SDS-PAGE, we conclude that the FTI ABT-100 suppressed the nuclear morphology phenotypes caused by the expression of *kuk*.

**Figure 4:**
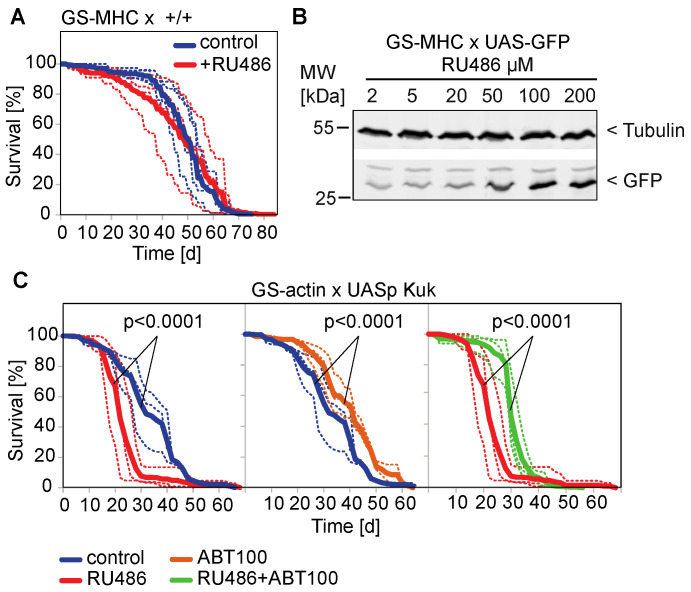
The FTI ABT-100 reextends experimentally induced shortened lifespan of *Drosophila* flies. Lifespan assays and Western blot with progenies and embryonic lysates of indicated crosses. Food was complemented with RU486 (200 µM) and ABT-100 as indicated. Shown are curves of individual assays (dashed lines) and average (solid lines). (**A**) Male progenies of cross GS-MHC x +/+. Four cages for each condition, n(–RU) = 291, n(+RU) = 277. The statistical significance for a difference of the average was *p* > 0.05. (**B**) Western blot for GFP and α–tubulin with extracts from thoraces of flies from cross GS-MHCxUAS-GFP, whose food was complemented with indicated concentration of RU486. (**C**) Male progenies of cross GS-actinxUAS-kuk. Three cages for each condition, n(–RU) = 217, n(+RU) = 297, n(–RU + ABT) = 363, n(+RU + ABT) = 363. The statistical significance was calculated by a log-rank test.

After five days of induced GFP-kuk expression, *kuk*-expressing Hela cells showed prominent H2AX staining, indicating the accumulation of unrepaired DNA damage (Figure 3C). GFP-kuk-expressing cells showed a strong reduction in HP1α staining after five days of induction (Figure 3D). In the Western blot analysis, a strong reduction in the Nurd complex component and heterochromatin marker RBBP4 was observed after five days of induction (Figure 3F, [4]). These results are consistent with our data obtained from the transiently transfected fibroblasts (Figure 1 and Figure 2, [29]). In summary, our cell culture experiments show that a set of farnesylated nuclear proteins and variants induce related nuclear phenotypes, which can be suppressed by FTIs and simvastatin. Thus, this cell culture system can serve as an assay for compounds or genes with an activity similar to FTIs and simvastatin.

### 3.3. The Farnesyl Transferase Inhibitor ABT-100 Reverses the Shortened Lifespan Induced by Kuk

The second part of our experimental system consists of an in vivo test with the lifespan as a readout. Whereas wild type *Drosophila* flies have a lifespan of two to three months depending on conditions, flies expressing *kuk* have a lifespan reduced by about half [29]. We employed these *kuk*-expressing *Drosophila* as an assay for the suppression of a shortened lifespan. We used the GeneSwitch system (GS system), which is induced by RU486, to drive *kuk* expression in adult *Drosophila*. Using the GS system allows for the minimization of problems of the genetic background as well as defects, which may arise during development [49,55]. In this system, the difference between the experimental and control condition is the presence or absence of the inducing agent RU486 in the food of adult flies with an identical genetic background and which were raised together as larvae and pupae without induction. Even high doses of RU486 did not affect the lifespan of flies (Figure 4A). Although we observed a variation in the survival curves between the individual cages, no striking difference in the average was observed. The concentration of 200 µM RU486 falls into the saturating range with our experimental settings. We observed about a 50% activation with a concentration of about 20 µM RU486 (Figure 4B, Supplemental Figure S1).

**Figure 5:**
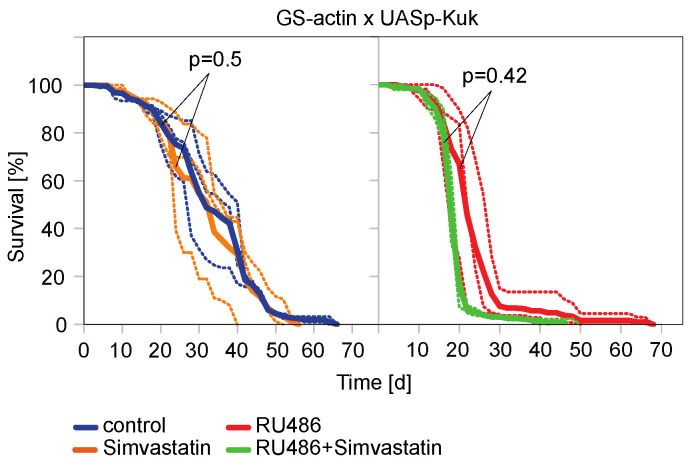
Simvastatin did not prolong experimentally induced shortened lifespan of *Drosophila* flies. Lifespan assays with flies expressing kuk under control of GS-actin driver line. Food was complemented with RU486 (200 µM) and Simvastatin as indicated. Shown are curves of individual assays (dashed lines) and average (solid lines). The statistical significance was calculated by a log-rank test. Three cages for each condition.

In agreement with the data of other GS-driver lines [29], the GS-actin line [48,56,57] driving ubiquitous UASp-kuk expression was able to decrease the lifespan (Figure 4C). When we treated non-induced GS-actin X UASp-kuk flies with ABT-100, we saw a significant extension of the mean lifespan compared to the untreated flies of the same genotype. Uninduced flies may express target genes in low levels as the GS system is prone to leakiness [56,58]. The treatment with ABT-100 significantly prolonged the mean lifespan of the induced flies compared to the induced flies which were not treated with ABT-100 (Figure 4C). Similarly, we tested the HMG-CoA reductase inhibitor Simvastatin in the lifespan assay. We did not observe any beneficial effect on the short lifespan of the *kuk*-expressing flies (Figure 5). In summary, we found that among the two drugs that were positive in the cell culture assay, only ABT-100 scored positive in the in vivo assay, as ABT-100 suppressed the shortened lifespan of the *kuk*-expressing flies.

**Figure 6:**
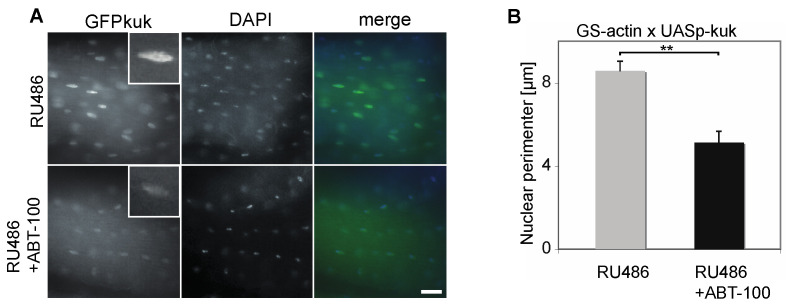
The FTI ABT-100 reduces nuclear perimeter size in *kuk*-expressing flies. (**A**) Fixed longitudinal muscle cells from males flies heterozygous for GS-actin and UASp-GFP-kuk stained for GFP (grey/green) and DAPI (grey/blue). Flies on food complemented with RU486 or RU486 + ABT-100 for five days. Insets show high magnification of a selected nucleus. Scale bar 10 µm. (**B**) Nuclear perimeters in longitudinal muscles. Samples from at least three flies each. n(RU) = 50, n(RU + ABT) = 62. Whiskers indicate standard error of the mean. Mann–Whitney U test: “**” indicates *p* < 0.001.

Finally, we tested whether the treatment with ABT-100 also affected the nuclear morphology in vivo. In *kuk*-overexpressing flies (GS-actin X UASp-GFP-kuk), we observed a prominent GFP-Kuk signal in the nuclei of the longitudinal flight muscles, which are a homogenous tissue easy to prepare (Figure 6A). Consistent with our previous data [29], the size of the nuclei was increased following induced *kuk* expression to an average nuclear perimeter of 8.6 ± 0.5 µm (±S.E.M., Figure 6B). After the treatment with ABT-100, the nuclear perimeter decreased significantly to 5.1 ± 0.5 µm, (±S.E.M., Figure 6B). These data suggest that ABT-100 ameliorates the nuclear phenotypes also in adult flies.

## 4. Discussion

Here, we report the development of an experimental system combining cell culture and adult Drosophila to test potential nuclear morphology- and lifespan-altering interventions. As an exemplary case, we confirm in our assays that the FTIs ABT-100 and FTI-277 and the HMG-CoA reductase inhibitor Simvastatin ameliorated the nuclear phenotypes induced by the farnesylated nuclear proteins Progerin, Kugelkern, or a Lamin B variant in the cell culture system. For the purpose of simplicity, we kept the scoring of the nuclear morphology and phenotypes on a qualitative level, although automated solutions for image analysis could be easily incorporated in a high throughput assay. On the organismal level, ABT-100 scored positive, in that the lifespan of the flies shortened by *kuk* expression was re-extended, whereas Simvastatin failed in the second test. Simvastatin is a specific inhibitor of the enzyme HMG-CoA reductase that catalyzes the conversion of HMG-CoA to mevalonate, which is the regulated entry step in the biosynthesis of cholesterol as well as in isoprene derivatives, including the farnesyl residue. Simvastatin failed to extend the lifespan in GS-actin X UASp-kuk-expressing flies, which may be explained by metabolic differences in sterol synthesis [59], as Drosophila and C. elegans are unable to synthesize sterols de novo [60,61]. However, the enzyme HMG-CoA reductase is present and isoprene and its derivatives can be synthesized by flies. Thus, the lack of activity of Simvastatin may be due to differences in the interaction of Simvastatin and the enzyme, inefficient uptake in the gut, or poor bioavailability, among other possibilities.

It is clear that FTIs affect a wide range of pathways and processes, most prominently small GTPases, such as RAS, besides the farnesylated proteins of the nuclear lamina. The application of FTIs to cells and an organism will impair these processes to a different degree depending on specific circumstances, such as the protein turnover or the sensitivity of the proteins to the loss of the farnesyl moiety. By comparing the induced and un-induced condition, we try to reduce complexity and aim to assign the phenotypes to the differences between the induced and un-induced condition. However, we cannot exclude the synergistic effects of other farnesylated proteins, as we do not directly measure the degree of farnesylation.

### 4.1. Premature Ageing Models

The study of molecular mechanisms underlying human ageing has been facilitated by studies on progeroid syndromes, including HGPS. In fibroblasts from HGPS patients, it has been shown that farnesyl transferase inhibitors (FTIs) restore nuclear shape abnormalities and reverse the changes in heterochromatin markers associated with Progerin accumulation. Accordingly, these compounds, originally developed as anticancer drugs, represent a therapeutic approach for HGPS. In ageing *C. elegans*, the FTI Gliotoxin was able to ameliorate nuclear morphology phenotypes and delay the age-dependent decline in locomotion. However, there was no effect on the lifespan in FTI-treated wild type worms [40]. In HGPS mouse models, FTIs reduce the incidence of nuclear deformities, improve their body weight, growth, and bone defects, and extend the lifespan of these mice [24,52,62]. These findings led to the clinical application of FTIs in HGPS patients [26,27].

Exposing adult *Drosophila* to ABT-100 reduced *kuk*-induced ageing-associated effects, like nuclear morphological aberrations and shortened the lifespan of the flies. ABT-100 may inhibit the farnesylation of Kugelkern, the expression of which is induced simultaneously with treatment with the drug, but also with other farnesylated proteins such as LaminDm0. For experimental reasons such as the similar genetic background, the lifespans are comparable between the induced and un-induced conditions. As the expression of farnesylated proteins is induced only in adult flies, the situation is different to a chronic expression throughout the life cycle, which is the case for genetic variants such as HGPS. Whether induced expression or chronic expression makes a difference is hard to estimate and may depend on the circumstances and the genes expressed. We could recapitulate the ageing-like phenotypes in *Drosophila* and successfully apply the therapeutic approach of FTIs. Thus, our findings show the feasibility of an assay for the identification of substances ameliorating nuclear phenotypes and re-extending the lifespan of short-lived flies expressing *kuk*.

### 4.2. Assay for Lifespan Altering Interventions

The molecular mechanisms of ageing are the focus of extensive investigations, providing hope for the discovery of the principles that govern this process, and novel ways to attenuate or delay it in humans. Research in the popular vertebrate genetic model systems mice and zebrafish has provided important insights into vertebrate ageing. However, both mice and zebrafish live for approximately three years or longer under laboratory conditions, which makes it very time-consuming and expensive to perform screens for genes or substances that prolong the lifespan. On the other hand, small and prolific organisms with a short lifespan, such as *C. elegans* and *Drosophila*, provide the basis for unbiased screens that identify and determine the functions of novel genes and substances that influence ageing in a physiological context. The small size and high fecundity of fruit flies has made them a favorite invertebrate model of developmental biologists. This has resulted in the detailed characterization of the *Drosophila* genome, the development of multiple mutant and transgenic phenotypes, and in molecular genetics, techniques allowing for the investigation of the intrinsic mechanisms underlying developmental or ageing-related events in this organism [63,64,65,66,67].

Specifically, the conditional gene expression system GeneSwitch provides several advantages for ageing studies, since it permits the temporal and tissue-specific control of the gene expression of a transgene in animals with identical genetic backgrounds [49,55,56,57,58,68]. Moreover, the GeneSwitch system provides powerful controls for the genetic background’s effects on lifespan, since control and gene-overexpressing animals have identical genetic backgrounds and differ only in the presence or absence of the expression-inducing drug. However, most GeneSwitch drivers tested are leaky under non-induced conditions ([56,58], Figure 4B), which implies that non-expressing controls cannot be obtained in strict terms. Furthermore, RU486 was recorded to affect food palatability and intake under certain conditions and to cause mating-dependent effects on lifespan in females [68,69,70].

Even the relatively short lifespan of the fruit fly (~6–12 weeks, depending on the conditions) makes the comprehensive investigations of ageing time-consuming. Thus, methods accelerating the screening of genetic and pharmacological agents for their lifespan-extending effects are needed. Here, we report the development of an assay in *Drosophila*, in which we shorten the lifespan of adult flies, thereby reducing the time needed to perform lifespan studies by about half. Through the over-expression of farnesylated nuclear proteins like Kuk, specific ageing-like phenotypes can be induced prematurely and the lifespan is shortened to less than four to five weeks. This shortening accelerates the ability to evaluate potential lifespan-altering interventions, thereby greatly facilitating drug or gene discovery.

## 5. Conclusions

Our experiments employing the FTI ABT-100 demonstrate that a combination of a cell culture test and population-scale Drosophila survivorship assays can be used as a test for pharmacologically active substances for lifespan-extending interventions. This combi-nation of first phasing down the number of candidate substances or genes that in the second step will be tested, could be used in time-consuming but relevant lifespan experiments.

## Figures and Tables

**Table 1:** Suppression of ageing-like phenotypes with ABT-100 treatment.

Nuclear Perimeter (µm)					
YFP-Progerin	Transfected	S.E.M.	Non-Transfected	S.E.M. ^(2)^	*p*-Value ^(1)^
control	61.79	±1.64	39.33	±1.26	<0.00001
ABT (3.6 µM)	39.05	±1.06	38.02	±0.87	n.s. ^(3)^
ABT (7 µM)	35.95	±1.19	36.00	±0.87	n.s.
Kuk					
control	42.17	±2.85	33.44	±2.85	0.021
ABT (3.6 µM)	30.94	±0.68	33.73	±0.65	n.s.
ABT (7 µM)	32.41	±0.72	33.04	±0.61	n.s.
GFP-LaminBDN					
control	60.13	±5.33	34.76	±1.27	0.00003
ABT (3.6 µM)	27.07	±1.14	31.75	±0.99	0.00032
ABT (7 µM)	33.49	±0.67	35.71	±1.10	n.s.

^(1)^ Mann–Whitney U-test, ^(2)^ standard error of the mean, ^(3)^ n.s. statistically not significant.

**Table 2:** Suppression of ageing-like phenotypes by Sim and FTI-277 treatment.

Nuclear Perimeter (µm)					
GFP-Progerin	Transfected	S.E.M.	Non-Transfected	S.E.M. ^(2)^	*p*-Value ^(1)^
control	53.48	±1.19	33.19	±0.82	<0.00001
Sim	38.69	±1.10	36.00	±0.85	0.043
FTI-277	42.66	±0.98	37.40	±1.44	n.s. ^(3)^
GFP-LaminBDN					
control	58.96	±18.19	34.83	±0.99	0.005
Sim	37.62	±2.67	33.57	±0.88	0.017
FTI-277	38.31	±4.83	36.63	±0.78	n.s.

^(1)^ Mann–Whitney U-test, ^(2)^ Standard error of the mean, ^(3)^ n.s. statistically not significant.

## Data Availability

All experimental data are available on request from J.G.

## Supplementary Materials

The following supporting information can be downloaded at: https://www.mdpi.com/article/10.3390/jdb11040040/s1, Figure S1: Source data to western blots.
