# Supplementary material for: Use of Farnesyl Transferase Inhibitors in an Ageing Model in Drosophila
Source: J Dev Biol. 2023 Oct 29;11(4):40. doi: 10.3390/jdb11040040 (PMC10660854; doi:10.3390/jdb11040040)
Supplement: Supplementary file 1 [file jdb-11-00040-s001.zip › jdb-2578830-supplementary.pdf]

Supplementary Data: Figure S1. Source data for Western blots

A: Source data to Figure 3E: KugelkernCS

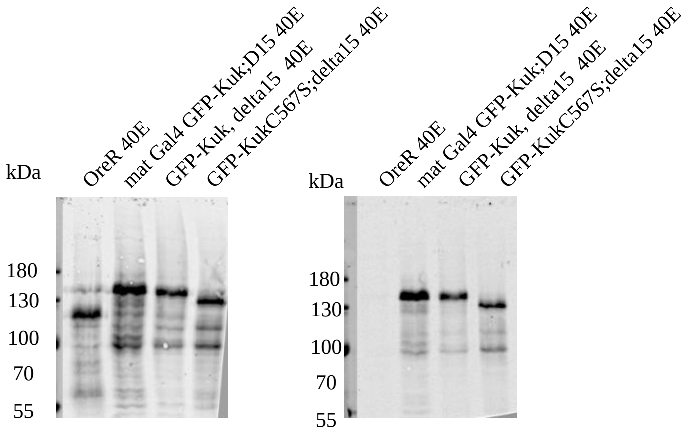

anti-Kuk (rabbit)
1: 10.000

anti-GFP (rabbit)
1: 6.000

6% SDS-Gel, Semidry-Transfer: 1,5h 60mA

B: Source data to Figure 3F: Kugelkern and RBBP4

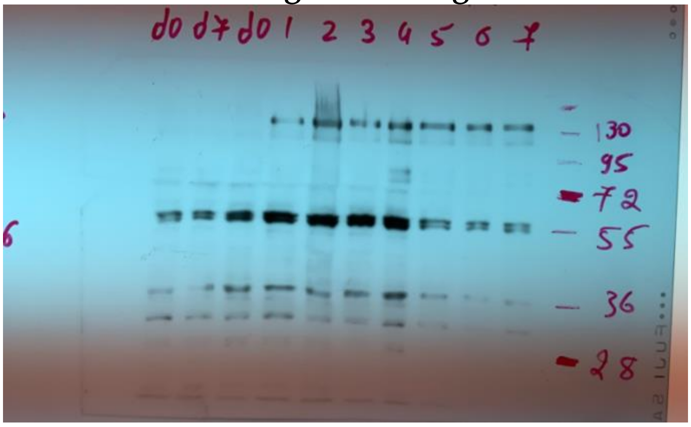

C: Source data to Figure 3F: pA414

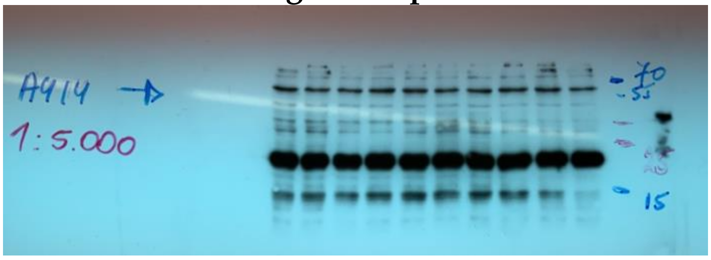

D Source data to Figure 3F: tubulin

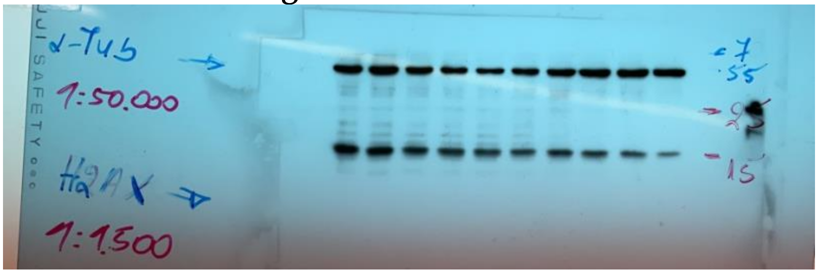

E: Source data to Figure 3G:

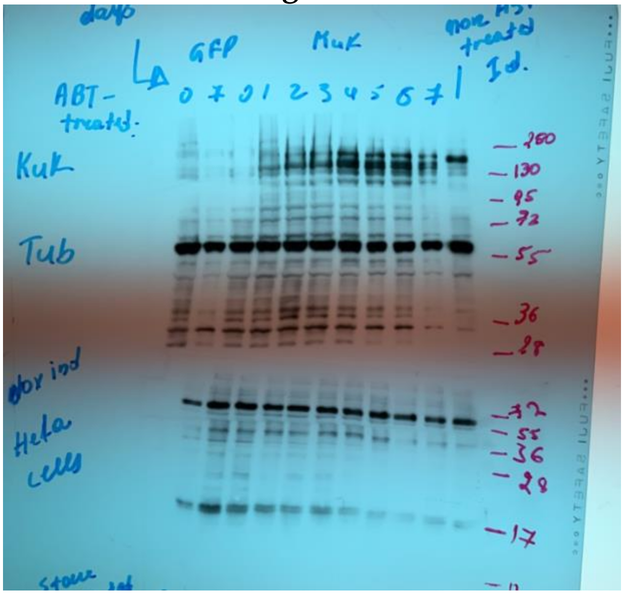

F: Source data to Figure 4B: GFP induction by RU

20170112 File: 4776

MHC-GS GAl4, UAS-GFP (10d Induction, 1,5 Thorax)

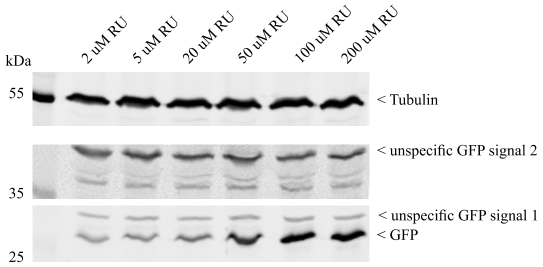

10 % SDS-Gel
Semidry-Transefer: 60 mA 1,5h
1st AB: anti-GFP (rabbit) (1:6.000) 2nd AB: anti-rabbit (goat, 800nm)
1st AB: anti-Tubulin (mouse) (1:75.000) 2nd AB: anti-rmouse (goat, 680nm)
